# Treatment of persistent allergic rhinitis via acupuncture at the sphenopalatine acupoint: a randomized controlled trial

**DOI:** 10.1186/s13063-017-2339-z

**Published:** 2018-01-11

**Authors:** Jiaoping Mi, Xinye Chen, Xiaoyan Lin, Jianling Guo, Hongtao Chen, Liao Wei, Haiyu Hong

**Affiliations:** 1 Department of Otolaryngology Head and Neck Surgery, the 5th Affiliated Hospital of Sun Yat-Sen University, Zhuhai, Guangdong 519020 People’s Republic of China; 2 Department of Otolaryngology, Guangdong Women and Children Hospital, Guangzhou, 511442 People’s Republic of China; 3 Department of Laboratory Medicine, the 5th Affiliated Hospital of Sun Yat-Sen University, Zhuhai, Guangdong 519020 People’s Republic of China

**Keywords:** Persistent allergic rhinitis (PAR), Acupuncture, Sphenopalatine acupoint (SPA), Nasal nerve adjustment

## Abstract

**Background:**

Allergic rhinitis is a common respiratory disease. Acupuncture is used to treat it in traditional Chinese medicine, and generally, the L120, ST2 and ST36 acupoints are selected in clinical practice. We report a new method of acupuncture at the sphenopalatine acupoint (SPA) for treatment of persistent allergic rhinitis (PAR). The effect of this treatment was investigated using two different needling depths. The efficacy of this treatment was associated with accurate stimulation of the sphenopalatine ganglion (SPG).

**Methods/Design:**

A total of 61 patients diagnosed with PAR were randomly allocated to either the acupuncture or the sham acupuncture group. The difference between the groups was the needle depth when acupuncture was administered, which was 50 mm and 20 mm. Alteration in total nasal symptom score (TNSS) was the primary outcome. Quality of life, medication dosages and adverse events were secondary outcomes, measured using the Rhinoconjunctivitis Quality of Life Questionnaire (RQLQ). Confidence assessment was performed to evaluate data from the treatment and follow-up periods.

**Results:**

Results were: (1) average TNSS in the treatment group was significantly lower than in the control group at week 4 (median and 25th and 75th percentiles were 5.00 (4.00, 7.00) and 8.00 (7.00, 10.00), respectively (*P* < 0.001)). However, scores in the two groups were not significantly different at week 12; (2) quality of life (RQLQ) was significantly improved at week 2 in the treatment group compared to the control group (scores of 35.47 ± 8.20 and 45.48 ± 8.84; *P* < 0.001); (3) during the follow-up period, the medication dosage in the treatment group was much lower than in the control group (3.64 ± 1.45 and 6.14 ± 2.34; *P* < 0.05); and (4) no adverse events were observed in either group during treatment.

**Conclusions:**

This pilot study revealed a profound effect of acupuncture at the SPA on prevention of PAR development. The TNSS in the treatment group (needle depth 50 mm), was significantly lower than in the control group (needle depth of only 20 mm). Our result demonstrates that performing acupuncture directly at the SPA to stimulate the SPG is an effective method to treat PAR.

**Trial registration:**

Acupuncture Clinical Trial Registry, AMCTR-OOR-16000014 and Chinese Clinical Trial Register, ChiCTR-IOR-16009211. Registered on 1 September 1 2016.

## Background

Allergic rhinitis (AR) is a chronic respiratory disease associated with a substantial health and psychological burden in patients due to its etiologically complex, prolonged disease course and high incidence. Persistent allergic rhinitis (PAR) has onset duration of more than 4 weeks per year and more than 4 days per week. Standard treatment for PAR includes allergen avoidance, effective symptomatic treatment, standardized immunotherapy, and patient health education [1]. Despite the clinical efficacy of these conventional treatments, there is concern over adverse effects. Treatment combining complementary and alternative medicine (CAM) improves clinical efficacy and reduces the incidence of adverse reactions [2].

Acupuncture, as one of the important therapies used in CAM, is based on the meridian theory, which targets certain specific acupoints to improve the body microenvironment and thus effectively treat certain diseases [3]. Recent research suggests that acupuncture may exert anti-inflammatory effects to prevent the development of AR via multiple neuroendocrine and immune network pathways [4]. Recent studies in the fields of neuroscience and immunology suggest an effect of neural reflexes in regulating the immune system, [3] especially in pathways involving the vagus nerve and macrophages [5].

However, acupuncture was not widely recommended for the treatment of AR in the past due to a lack of solid clinical and experimental evidence supporting its efficacy [1]. Since 2015, accumulated evidence, especially from multicenter randomized controlled clinical trials, has demonstrated the efficacy of acupuncture in treating PAR [6–9]. As a result, acupuncture is now listed on the AR guidelines in the USA [10].

The sphenopalatine acupoint (SPA) is located in the cheek [11, 12] near ST7 (Xiaguan) [13], which is generally used as the needling point for local nasal anesthesia. Technically, the needle is inserted through this acupoint to reach the pterygopalatine fossa (PPF) [14] to stimulate the sphenopalatine ganglion (SPG) and the other nasal nerve [15], increase nasal ventilation and decrease the glandular secretion. The use of acupuncture at the SPA to treat AR was initially reported in 1990 [12]. The author has long been engaged in acupuncture research and has proved its clinical efficacy in the treatment of AR [16].

The curative effect of acupuncture on PAR is mediated by anti-inflammatory effects via the regulation of the interaction between the vagus nerve and macrophages. These generic anti-inflammatory effects directly prevent the development of AR [8]. As the SPG is a parasympathetic ganglion capable of nasal function adjustment, we hypothesized that acupuncture at the SPA may lead to better efficacy because SPA stimulation can directly act on the SPG and can consequently yield an improvement in nasal function. The aim of this study was to determine the effectiveness of acupuncture at the SPA for preventing PAR and to compare the efficacy and adverse effects of acupuncture at different needle depths in a randomized controlled trial.

## Methods/Design

### Study design

This randomized, controlled and double-blinded study was designed as a first step to evaluate the efficacy of acupuncture for PAR treatment. The study was conducted at the Ear, Nose and Throat (ENT) Department of the 5^th^ Affiliated Hospital of Sun Yat-Sen University (SYSU) in China. The study was sequentially conducted as follows.

During a run-in period of 14 days prior to randomization, the patients stopped taking medication and were instructed on how to use the score card. During the following week, the patients recorded symptom scores every day to establish an individualized 7-day baseline score. At the end of the run-in period, participants were randomized into either the treatment group or control group by a computer-generated random number allocation scheme.

The treatment lasted 4 weeks, and acupuncture was performed twice a week. Then, patients underwent a 12-week follow-up period. The interval from the beginning of treatment to the end of follow up was 16 weeks.

The study protocol was approved by the Ethics Committee of the 5th Affiliated Hospital of SYSU. Signed informed consent forms (ICFs) were obtained from participants before they were assigned into the study groups. The study was conducted in accordance with the principles of the Declaration of Helsinki (2004) and in accordance with the Medical Research Involving Human Subjects Act (WMO). The study was registered in the Acupuncture Clinical Trial Registry (AMCTR-OOR-16000014) and in the Chinese Clinical Trial Register (ChiCTR-IOR-16009211) on 1 September 2016.

### Subjects

Patients suffering from PAR were selected from the ENT Department between June 2010 and March 2015. The inclusion criteria were (1) patients suffering from moderate-to-severe PAR, aged between 18 and 65 years, with a positive skin prick test to house dust mites (ALK reagent) according to the Allergic Rhinitis and its Impact on Asthma criteria (ARIA, 2008); (2) patients diagnosed with the disease for at least 1 year and whose sleep quality was affected during the onset period; (3) patients of any gender or ethnicity; (4) patients who were using female contraception; and (5) patients who signed the ICF. The exclusion criteria were (1) patients younger than 18 years or older than 65 years; (2) pregnant or breastfeeding women; (3) patients with nasal polyps, sinusitis, or an obvious deviated nasal septum; (4) patients with long-term use of corticosteroids or immunosuppressive agents; (5) patients with an irregular lifestyle; (6) patients with cerebrovascular, lung, liver, kidney, or cardiovascular diseases; and (7) patients who were unable to comply with the follow-up requests.

### Randomization and double blinding

**Fig. 1:**
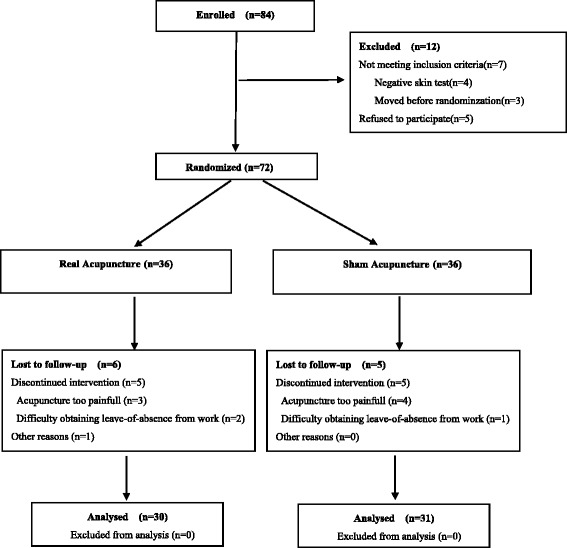
Consolidated Standards of Reporting Trials (CONSORT) flow diagram describing participant allocation in this study

Based on the order of admission to the outpatient clinic, the patients were randomly allocated at a 1:1 ratio into either the treatment (real acupuncture group (RAG) (*n* = 42)) or the control (sham acupuncture group (SAG) (*n* = 42)) groups using a computer-generated random sequence at the Institute of Medical Information Technology, Biometry and Epidemiology, SYSU, China. Excluded subjects (*n* = 12) were withdrawn from the study as they failed to meet the eligibility criteria. Six patients in the RAG were lost during follow up, while five patients in the SAG were lost. A total of 61 patients completed the study, including 30 in the RAG and 31 in the SAG (Fig. 1). The allocation to the treatment or control group will then be performed using sealed consecutively numbered envelopes prepared by the trial team. After randomization, the group assignments were concealed and sealed sequentially into numbered light-proof envelopes.

The researchers were divided into two groups. The ENT physicians were responsible for the diagnosis and therapeutic assessment of the patients. The acupuncturists were responsible for the acupoint localization and acupuncture performance. Once the patient met the inclusion criteria, the person in charge of the envelopes gave the specified envelope to the acupuncturist. To preserve masking, only the acupuncturists had access to the treatment allocation. The acupuncture practitioners were informed of this due to the nature of the intervention, but they were asked to not communicate with the participants or assessors about the treatment procedures and responses. In this study, neither the patients nor the ENT physicians were aware of the list of patients in the RAG and SAG before data decoding.

### Intervention

**Fig. 2:**
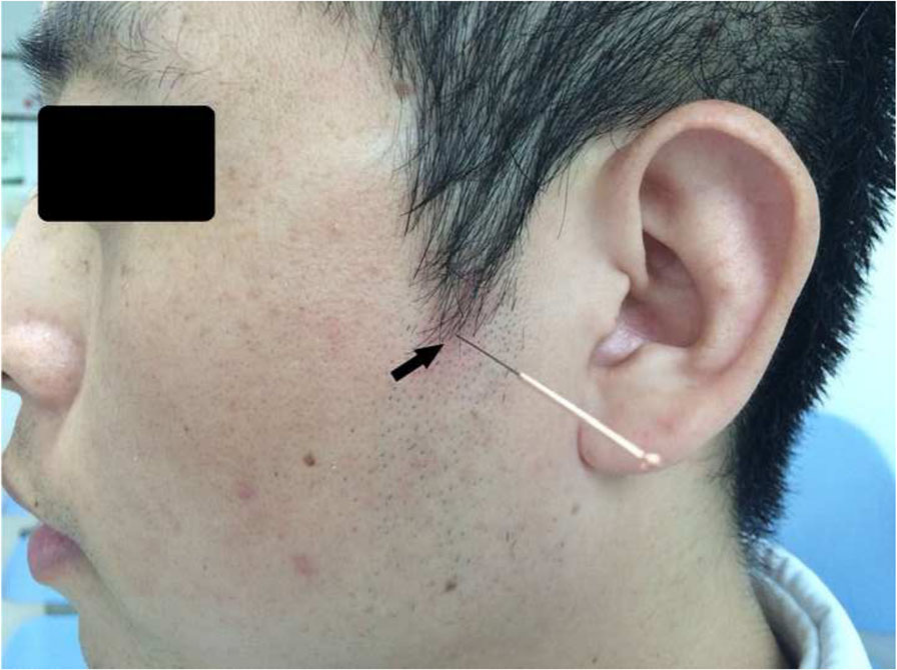
Acupuncture needle point. The black arrow shows the Die-e acupoint, which is located under the zygomatic arch between the coronoid process and the mandibular condyle. Acupuncture on the Die-e point at a needling depth of 50 mm and 20 mm in the real acupuncture group and in the sham acupuncture group, respectively

The intervention was conducted as follows: (1) based on earlier reports [11, 12] and the authors’ clinical experiences [16], the SPA was located under the zygomatic arch and between the coronoid process and mandibular condyle; (2) the GLOBAL acupuncture needles measuring 0.3*75 mm were purchased from Suzhou Acupuncture Supplies, Ltd. (Suzhou Food and Drug Administration 2008; batch/lot number: 2270318, Suzhou, Jiangsu Province, China); (3) patients were placed in the sitting position, and the acupoint area was disinfected twice with iodine. The acupuncturist placed his/her left index finger immediately above the acupoint, held the needle between his/her right thumb and index finger, and quickly pierced the skin of the patient. The slow inward pressure and twisting of the needle introduced a sensation of De Qi, a radiating numb sensation and acid bilge feeling in the upper teeth. The acupuncturist then twisted the needle repeatedly at a speed of 3–5 r/s without the use of an electrical or laser instrument. While the needle was pulled out, sterilized cotton was pressed to the cheek for 5 minutes (Fig. 2); (4) the treatment time period was 4 weeks, and needling was performed twice a week; (5) the needle depths were approximately 50 mm in the RAG and 20 mm in the SAG.

### Main outcome

The main outcome of this study was the total nasal symptom score (TNSS) [17]. The primary efficacy indicators included four common symptoms of AR: rhinorrhea, nasal obstruction, sneezing, and nasal itching. The sum of the TNSS, including the four symptom scores, was calculated. Each symptom was scored from 1 to 3 according to the severity. The following scores were used. Sneezing: continuous sneezing 3–5 times per day was scored as 1, sneezing 6–10 times per day was scored as 2, and sneezing more than 11 times per day was scored as 3. Rhinorrhea: fewer than 4 times per day was scored as 1, 5–9 times per day was scored as 2, and more than 10 times per day was scored as 3. Nasal obstruction with inhalation was scored as 1, intermittent nasal obstruction was scored as 2, and breathing by mouth during the day was scored as 3. Intermittent nasal itching was scored as 1, tolerant formication was scored as 2, and unbearable formication was scored as 3.

### Secondary outcomes

The secondary outcome indicators included the evaluation of quality of life and of medication dosage during follow up. Quality of life was scored according to the Rhinoconjunctivitis Quality of Life Questionnaire (RQLQ) [18]. The main evaluation parameters were non-nasal/ocular symptoms and behavior and emotional function. In the case of unbearable nasal symptoms, the patient was allowed to take loratadine (10 mg/pill) once per day. Each pill was scored as 1 in the evaluation table.

### Confidence assessment

A 7-day baseline was established during the run-in period, which included the nasal symptom scores and quality of life before the treatment. An efficacy evaluation of the patients was performed by the physicians every 2 weeks. In addition to recording their daily score cards during the treatment period, the patients received an evaluation list every 4 weeks during the follow-up period [3–5]. The observation included the main outcome and secondary outcome indicators, which were recorded on the week of 12 August 2016.

### Statistical analysis

The statistical analysis was performed using the SPSS statistical software system (SPSS Inc., Chicago, IL; version 13.0). Data are represented as the mean and standard deviation. Nonparametric tests were used to compare pre-treatment and post-treatment data in the groups that were not normally distributed. Intra-group comparisons were performed using the paired *t* test or the Wilcoxon rank-sum test. Two-sided *P* values <0.05 were considered statistically significant.

## Results

### Patient demographics

A total of 61 patients were divided into two groups (the RAG and SAG). No significant differences were observed between these two groups in the history of PAR and the RQLQ score (Table 1). No significant difference was found in the variations in the TNSS or RQLQ between the two groups before treatment (Table 2).

**Table 1:** Comparison of participant characteristics in the two groups

	Real acupuncture group	Sham acupuncture group	*P* value
	(*N* = 30)	(*N* = 31)	
Age (years)	35.87 ± 10.36	35.97 ± 9.15	0.550
Male/female, number of participants	16/14	17/14	0.521
History of PAR	49.03 ± 29.84	51.17 ± 32.39	0.907
RQLQ score	45.93 ± 8.67	49.16 ± 7.87	0.133
Sleep	7.9 ± 3.34	8.00 ± 4.48	0.922
Non-nasal/eye	18.83 ± 4.43	20.81 ± 4.44	0.086
Behavior	12.00 ± 3.00	12.1 ± 3.36	0.905
Emotional function	7.23 ± 2.5	8.3 ± 2.15	0.081

Values are expressed as mean ± SD unless stated otherwise

*PAR *persistent allergic rhinitis, *RQLQ* Rhinoconjunctivitis Quality of Life Questionnaire

*P* > 0.05 indicates there was no statistically significant difference

**Table 2:** Baseline 14-day nasal symptom scores in the two groups (median (25^th^ percentile, 75^th^ percentile))

	Real acupuncture group	Sham acupuncture group	*P* value
	(*N* = 30)	(*N* = 31)	
Nasal obstruction	2.00 (2.00, 3.00)	3.00 (2.00, 3.00)	0.521
Rhinorrhea	2.00 (2.00, 3.00)	2.00 (2.00, 3.00)	0.759
Sneezing	3.00 (2.00, 3.00)	3.00 (2.00, 3.00)	0.398
Nasal itch	2.00 (1.00, 2.00)	1.00 (1.00, 2.00)	0.301
TNSS	9.00 (7.75, 10.00)	9.00 (7.00, 10.00)	0.618

The Mann-Whitney U test was used to compare the real acupuncture group with the sham acupuncture group

*TNSS* total nasal symptom score

*P* > 0.05 indicates no statistically significant differences

### TNSS

Nasal obstruction and sneezing improved at week 2 in the RAG compared to that in the SAG (*P* < 0.001). All the indicators improved at the end of the treatment. Compared to the patients in the SAG, the symptoms of nasal obstruction and sneezing improved in the RAG within 4 weeks of the post-treatment period (*P* < 0.001), and the improvement in nasal obstruction continued for 8 weeks after treatment in the RAG (*P* < 0.05) (Table 3).

**Table 3:** Changes from baseline in the 14-day nasal symptom scores (median (25^th^ percentile, 75^th^ percentile))

	Real acupuncture group	Sham acupuncture group	*P* value
	(*N* = 30)	(*N* = 31)	
After treatment (week 2)			
Nasal obstruction	2.00 (1.00, 2.00)*	2.00 (2.00, 3.00)	<0.001
Rhinorrhea	2.00 (2.00, 2.00)*	2.00 (2.00, 3.00)	NS
Sneezing	1.50 (1.00, 2.00)*	2.00 (2.00, 3.00)	<0.001
Nasal itch	1.00 (1.00, 3.00)*	1.00 (0.00, 2.00)	NS
TNSS	6.00 (5.75, 7.00)*	7.00 (7.00, 10.00)	<0.001
Follow up (week 4)			
Nasal obstruction	1.00 (1.00, 2.00)*	2.00 (2.00, 3.00)	<0.001
Rhinorrhea	2.00 (1.00, 2.00)*	2.00 (2.00, 3.00)	<0.01
Sneezing	1.00 (1.00, 2.00)*	3.00 (2.00, 3.00)	<0.001
Nasal itch	1.00 (0.00, 1.00)*	2.00 (1.00, 2.00)	<0.01
TNSS	5.00 (4.00, 7.00)*	8.00 (7.00, 10.00)	<0.001
Follow up (week 8)			
Nasal obstruction	2.00 (1.00, 2.00)	2.00 (2.00, 3.00)	<0.001
Rhinorrhea	2.00 (2.00, 3.00)	2.00 (2.00, 3.00)	NS
Sneezing	2.00 (1.00, 2.00)*	3.00 (2.00, 3.00)	<0.01
Nasal itch	2.00 (1.00, 2.00)	1.00 (1.00, 2.00)	NS
TNSS	7.00 (6.00, 8.00)*	8.00 (7.00, 10.00)	<0.01
Follow up (week 12)			
Nasal obstruction	2.00 (1.00, 2.00)	2.00 (2.00, 3.00)	<0.05
Rhinorrhea	2.00 (2.00, 3.00)	3.00 (2.00, 3.00)	NS
Sneezing	3.00 (2.00, 2.00)	3.00 (2.00, 3.00)	NS
Nasal itch	2.00 (1.00, 2.00)	2.00 (1.00, 2.00)	NS
TNSS	8.00 (7.00, 10.00)	9.00 (8.00, 9.00)	NS
Follow up (week 16)			
Nasal obstruction	2.00 (2.00, 3.00)	2.00 (2.00, 3.00)	NS
Rhinorrhea	2.00 (2.00, 2.25)	2.00 (2.00, 3.00)	NS
Sneezing	2.00 (2.00, 3.00)	2.00 (2.00, 3.00)	NS
Nasal itch	1.00 (1.00, 2.00)	1.00 (1.00, 2.00)	NS
TNSS	8.00 (7.00, 10.00)	9.00 (1.00, 10.00)	NS

*NS* no significant difference

*P* values are for comparison of the real acupuncture group and the sham acupuncture group

*Statistically significant compared to the baseline score when assessed against the least significant difference (α: 0.05/3 = 0.0167)

**Fig. 3:**
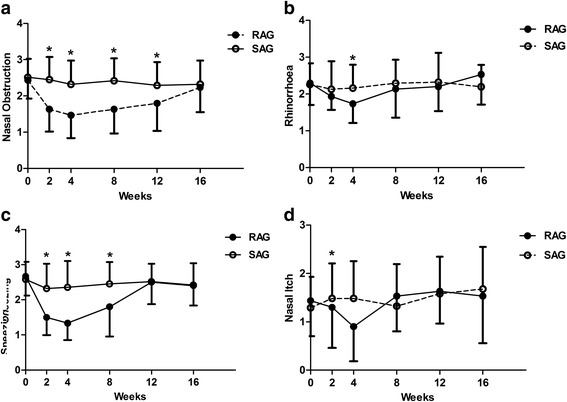
Changes in the symptoms of TNSS, such as nasal obstruction (**a**), rhinorrhea (**b**), sneezing (**c**), and nasal itch (**d**), in the RAG and SAG. Asteriks indicate the time points in which there were significant differences between the two groups (*P* < 0.05). Only the upper or lower standard deviation is shown for clarity. *TNSS* total nasal symptom score; *RAG* real acupuncture group; *SAG* sham acupuncture group

According to the efficacy curves (Fig. 3a-e), the TNSS in the RAG was significantly lower than in the SAG during the treatment and follow-up period. Both the TNSS and individual entry scores in the RAG were lowest at week 4. This efficacy was maintained until week 8. No significant differences were found between the two groups at weeks 12 and 16 (Fig. 3).

### Quality of life

The sleep scores showed no differences between the RAG and SAG, but the non-nasal/eye and behavior and emotion function scores showed significant differences between the two groups at weeks 2 and 4 (*P* < 0.05). The non-nasal/eye and emotion function scores showed significant differences between the RAG and SAG at week 8 (*P* < 0.01 and *P* < 0.05, respectively) (Table 4).

**Table 4:** RQLQ scores for sleep, non-nasal/eye symptoms, behavior and emotional function between the two groups (mean ± SD)

	Real acupuncture group	Sham acupuncture group	*P* value
	(*N* = 30)	(*N* = 31)	
Sleep	6.30 ± 3.10	7.06 ± 4.45	NS
Non-nasal/eye	14.17 ± 4.07	18.65 ± 4.90	<0.001
Behavior	9.27 ± 2.98	12..7 ± 3.36	<0.01
Emotional function	5.73 ± 2.42	7.71 ± 2.45	<0.001
Sleep	5.93 ± 3.34	7.36 ± 4.56	NS
Non-nasal/eye	12.93 ± 3.53	20.74 ± 5.37	<0.001
Behavior	8.17 ± 3.02	12.20 ± 3.65	<0.001
Emotional function	4.03 ± 1.61	7.13 ± 2.91	<0.001
Sleep	7.47 ± 3.76	9.00 ± 4.22	NS
Non-nasal/eye	15.77 ± 4.67	20.13 ± 5.49	<0.01
Behavior	11.77 ± 3.10	12.45 ± 2.82	NS
Emotional function	5.73 ± 2.00	7.26 ± 2.65	<0.05
Sleep	8.07 ± 2.89	9.00 ± 4.22	NS
Non-nasal/eye	18.27 ± 4.65	20.13 ± 5.49	NS
Behavior	11.27 ± 2.80	12.45 ± 2.81	NS
Emotional function	6.60 ± 2.30	7.26 ± 2.65	NS
Sleep	8.07 ± 3.36	9.00 ± 4.17	NS
Non-nasal/eye	18.07 ± 4.77	20.48 ± 4.93	NS
Behavior	10.93 ± 3.02	12.00 ± 3.63	NS
Emotional function	7.23 ± 2.34	7.62 ± 2.78	NS

*NS* no significant difference, *RQLQ* Rhinoconjunctivitis Quality of Life Questionnaire

*P* values are for comparison of values in the real acupuncture group and sham acupuncture group

**Fig. 4:**
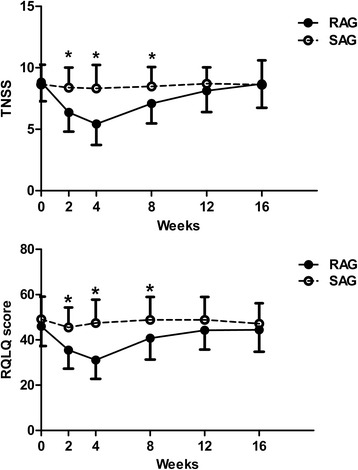
The changes in the TNSS and RQLQ in the RAG and SAG. Asteriks indicate the time points with significant differences between the two groups (*: P < 0.05). Only the upper or lower standard deviation is shown for clarity. *TNSS* total nasal symptom score. *RQLQ* Rhinoconjunctivitis Quality of Life Questionnaire; *RAG* real acupuncture group; *SAG* sham acupuncture group

The RQLQ score in the RAG was improved at week 2 compared to that in the SAG (24.37 ± 7.50 vs. 49.60 ± 8.50; *P* < 0.001). The RQLQ score in the RAG was lower than that in the SAG at week 4 of the post-treatment period (40.73 ± 9.46 vs. 48.84 ± 10.13; *P* < 0.01), but this difference was not present at weeks 12 and 16 (*P* > 0.05) (Fig. 4).

### Medication during follow up

During the follow-up period, the patients in both groups were allowed to take one loratadine pill once a day, which was scored correspondingly in the table. The use of medication was observed at week 12 in the SAG and at week 16 in the RAG. Significant differences were observed in the accumulated dosage between the two groups (3.64 ± 1.45 vs. 6.14 ± 2.34; *P* < 0.05).

### Safety analysis

No adverse events, such as local hematoma, were reported in either group during the clinical trial. In addition, no patient suspended the treatment due to pain.

## Discussion

In traditional Chinese medicine (TCM), PAR is categorized as a deficiency syndrome because of defective “Zang-Fu” (body organ) function, weak immune defenses and susceptibility to exogenous factors, such as the cold, wind and dampness [19]. Acupuncture is based on the meridian theory and has a long history of treatment of AR. Based on the principle of “cure along unobstructed meridians”, multiple acupoints were chosen for this complaint, of which the most commonly selected acupoints are L120, ST2 and ST36 [7, 20]. The mechanism of action involves Qi and blood regulation and Yin-Yang balance.

Acupoints are regarded as Qi and as blood transmission surface nodes in the terminal point of “Zang-Fu” organs and along the meridians. From a modern viewpoint, the acupoints are areas of dense nerve terminals and nerve stems. An acupoint is essentially a bundle of nerves and blood vessels in the bone, muscles and fascia and cerebral spinal nerves in the shallow areas of the intersection with the body axis [21]. The SPA is a recently discovered acupoint that is used in the treatment of nasal disease [12]. The needle is inserted at the site directly contacting the PPF and at a depth of 46 mm [11]. The SPG is a parasympathetic ganglion in the PPF [15] that contains branches of the trigeminal nerve and sympathetic and parasympathetic fibers and regulates nasal function as the trigger point or sensitive area.

The SPA is different from the traditional acupoint in that it is a single acupoint rather than a grouped acupoint. As the acupoint is located in the bone gap, a needle is used to access the pathway and obtain a sensation of “De Qi”, therefore, only acupuncture can achieve curative effects.

PAR is an IgE-mediated chronic inflammatory nasal disease. Allergies cause inflammation of the nasal mucosa, sensitization of the nasal sensory nerve, activation of the parasympathetic nervous system, and inhibition of sympathetic nerve function. The dysfunction of the autonomic nervous system is an important trigger for the onset of nasal diseases and escalation of clinical symptoms [22].

The efficacy of modern acupuncture is related to its anti-inflammatory effects via a complex neuroendocrine and immunological network [4]. A negative feedback loop between the autonomic nervous system and innate immunity modulates the body’s immune response [9].

The effect of acupuncture at the SPA may be mediated via the autonomic nervous system and innate immunity. Acupuncture affects nasal function by the regulation of neural reflexes in the immune system [9]. Experimental evidence suggests that the electrical stimulation of the vagus nerve inhibits the activation of macrophages and reduces the production of pro-inflammatory cytokines such as TNF, IL-1 beta, IL-6, and IL-18 [10]. Insertion of the needle at the PPF affects neural function through the SPG, attenuates the sensitivity of sensory nerves, balances the autonomic nervous function in the nose and downregulates the sensitization of the central nervous system. Wang conducted a series of objective tests to confirm the change in concentration of inflammatory factors and improvement in nasal ventilation with acupuncture at the SPG [23], which may reasonably explain why the treatment can reduce nasal hyper-responsiveness and the frequency of onset of symptoms.

Dust mites are the most common allergens in Southern China [24]. The allergen density in the air constantly varies in the subtropical climate. Patients with PAR are allergic to dust mites, and the onset of the disease is perennial. The investigators of this study are experts who are trained in TCM. All acupuncture therapists were certified, ensuring the quality of treatment in this study [8].

Based on the clinical data, the observations were divided into three periods using the author’s method to maintain quality control [6]. When a patient under medication visited the ENT physicians, he/she was immediately requested to cease antihistamine medication and to start using the symptom score card record during the second week. Loratadine is fully metabolized within 7 days. The therapeutic effect of acupuncture was obtained within 10 weeks [6], and the effect was still present after 12 weeks in the RAG [12]. Although medication was allowed during the follow-up period, the dosage in the RAG was significantly lower than that in the SAG. Therefore, it did not affect the treatment outcome.

The SPG in the PPF modulates nasal inhalation, nasal sensation, vasomotor responses of the nasal mucosa, and glandular secretions. Disorders in nerve function lead to nasal symptoms, among which nasal congestion is a common symptom. Fleckenstein’s study showed significant effects of acupuncture treatment compared to a sham treatment on the nasal sickness score for symptoms of vasomotor rhinitis [25]. Our study showed that acupuncture relieved symptoms of nasal congestion and runny nose. However, significant differences between the two symptoms were observed. The relief of nasal congestion occurred earlier than that of runny nose, which is likely due to the different sensitivities to neurotransmitters of the target organs. Blood vessels are more sensitive to nerve responses than glandular tissues, [26] and the nasal symptoms were more significantly relieved in the RAG than in the SAG. Nasal congestion could be relieved immediately in some of the patients when needling was performed at the SPA. The nerve reflex may be related to the efficacy time via the regulation of the vagus nerve and macrophages.

Evaluation of quality of life was performed using the RQLQ, which contains four major entries. The sleep entry was the most important factor because nasal congestion mostly affects sleep. The patients reported their quality of life to be much improved after the treatment compared to that before the treatment.

## Conclusions

The onset of PAR is related to allergy and nasal neurological disorders. Acupuncture at the SPA is an effective treatment for PAR, as it may ameliorate nasal symptoms, improve quality of life, and reduce the dosage of medicine. The responding area of the SPA is located at the PPF. By stimulating the SPG and trigeminal nerve, acupuncture lowered the sensitivity of the nasal sensory nerves, balanced the autonomic nervous system, downregulated the central nervous system sensation, and attenuated the overall nasal hyper-responsiveness. The SPA is a single acupoint that is used to treat PAR, and repeated acupuncture sessions produced no adverse effects, thus strengthening its appeal and potential clinical application. The results from this study were obtained from a small sample. Therefore, further validation of these results with larger-scale, multicenter clinical trials using more objective indicators could be decisive. Our study results are reasonable due to the mechanistic interaction between the reflexive nerve loop and inflammation-related immune cells.

## Abbreviations

AR: Allergic rhinitis
CAM: Complementary and alternative medicine
ENT: Ear, nose and throat
IL: Interleukin
ICFs: Informed consent forms
PAR: Persistent allergic rhinitis
PPF: Pterygopalatine fossa
RAG: Real acupuncture group
RQLQ: Rhinoconjunctivitis Quality of Life Questionnaire
SAG: Sham acupuncture group
SPA: Sphenopalatine acupoint
SPG: Sphenopalatine ganglion
TNF: Tumor necrosis factor
TNSS: Total nasal symptom score
